# Iron–Salen Complex and Co^2+^ Ion‐Derived Cobalt–Iron Hydroxide/Carbon Nanohybrid as an Efficient Oxygen Evolution Electrocatalyst

**DOI:** 10.1002/advs.201900117

**Published:** 2019-04-15

**Authors:** Jian Du, Guoquan Liu, Fei Li, Yong Zhu, Licheng Sun

**Affiliations:** ^1^ State Key Laboratory of Fine Chemicals DUT‐KTH Joint Education and Research Center on Molecular Devices Dalian University of Technology Dalian 116024 China; ^2^ Department of Chemistry KTH Royal Institute of Technology Stockholm 10044 Sweden

**Keywords:** CoFe hydroxides, electrocatalysts, iron–salen complexes, molecular precursor, oxygen evolution reaction

## Abstract

Metal–salen complexes are widely used as catalysts in numerous fundamental organic transformation reactions. Here, CoFe hydroxide/carbon nanohybrid is reported as an efficient oxygen evolution electrocatalyst derived from the in situ formed molecular Fe–salen complexes and Co^2+^ ions at a low temperature of 160 °C. It has been evidenced that Fe–salen as a molecular precursor facilitates the confined‐growth of metal hydroxides, while Co^2+^ plays a critical role in catalyzing the transformation of organic ligand into nanocarbons and constitutes an essential component for CoFe hydroxide. The resulting Co_1.2_Fe/C hybrid material requires an overpotential of 260 mV at a current density of 10 mA cm^−2^ with high durability. The high activity is contributed to uniform distribution of CoFe hydroxides on carbon layer and excellent electron conductivity caused by intimate contact between metal and nanocarbon. Given the diversity of molecular precursors, these results represent a promising approach to high‐performance carbon‐based water splitting catalysts.

Electrochemical water splitting is as a promising approach for sustainable hydrogen production.^1^, ^2^, ^3^, ^4^, ^5^, ^6^ Since the efficiency of overall water splitting is restricted by the sluggish kinetics of oxygen evolution reaction (OER) due to multiple proton/electron transfer processes,^7^, ^8^, ^9^ it is important to develop efficient OER electrocatalysts with low overpotential and high durability. To date, the state‐of‐the‐art OER catalysts are relying on noble metals, their large‐scale application is impeded by high cost and scarcity.^10^, ^11^, ^12^, ^13^, ^14^, ^15^ Therefore, earth‐abundant transition metal‐based hydroxides and oxides as alternative catalysts have attracted extensive attention.^16^, ^17^, ^18^, ^19^, ^20^, ^21^, ^22^, ^23^ However, severe aggregation of metal centers and intrinsically poor electronic conductivity limit the performance of these low‐cost materials in OER.

To alleviate these disadvantages, metal (hydr)oxides have been hybridized with carbon materials such as graphene,^24^ carbon nanotubes (CNT),^25^ and carbon fibers^26^ to improve the conductivity and enlarge the active surface of electrodes. In this regard, metal‐organic frameworks (MOFs) are ideal precursors for porous carbon‐supported OER catalysts owing to the strong interaction between metal active sites and molecular backbone‐derived nanocarbons.^27^, ^28^, ^29^ However, the MOF‐derived hybrids with suitable graphitic structures are normally obtained at high temperatures (> 800 °C) due to the high chemical and thermal stability of ordered frameworks.^30^, ^31^ The harsh conditions used for carbonization of the porous coordination polymers like MOFs, in principle, could be avoided by using molecular complexes with discrete structures as the precursors. To date, only one related example has appeared in literature.^32^ In that study, Xu and co‐workers described an in situ formed OER electrocatalyst consisting of interconnected NiFe–LDH and carbon nanodomains by solvothermal reaction of Ni^2+^, Fe^3+^, and 2‐mercapto‐5‐nitrobenzimidazole (MNBI) in *N*,*N*‐dimethylformamide (DMF). MNBI as the carbon source was presumed to interact with metal cations though a precise coordination chemistry in solution is unclear.

To fully realize this concept and develop a facile method for preparing electrocatalyst, we report here the solvothermal fabrication of a bimetallic CoFe hydroxide/carbon composite (CoFe/C) with an easily‐accessible molecular Fe–salen (salen = bis(salicylidine)ethylenediamine) complex as precursors at a temperature as low as 160 °C. The resulting hybrid material exhibited high OER performance in alkaline media with a low overpotential of 260 mV for achieving a current of 10 mA cm^−2^, favorable reaction kinetics and outstanding long‐term durability.

Metal–salen complexes are famous for their widespread application in a variety of catalytic reactions such as polymerization and oxidation.^33^, ^34^, ^35^ To the best of our knowledge, there is still no report on carbon–metal hybrid derived from a metal–salen complex. We envisioned that the molecular identity of metal–salen complexes might benefit a homogenous distribution of the resulting metal‐based nanoparticles and intimate contact between metal and carbon derived from organic ligands, resulting in enhanced exposure of catalytic active sites and higher electron conductivity. In addition, the ease of synthesis and diversity of salen ligands allow to systemically optimize the properties of electrocatalysts.

The preparation process is illustrated in **Scheme** 1. The salen ligand, *N*,*N*′‐bis‐(2,3‐dihydroxybenzylididene)‐o‐phenylenediamine (salen‐1), was simply synthesized by aldehyde‐ammonia condensation reaction (see the Supporting Information). Fe,Co hydroxides decorated nanocarbon (Fe, Co/C) electrocatalyst was fabricated by an one‐step, in situ solvothermal reaction of salen‐1, Fe(NO_3_)_3_ and Co(NO_3_)_2_ at a ratio of 1:1:2 in DMF at 160 °C. The as‐prepared product was obtained as a black precipitate.

**Scheme 1:**
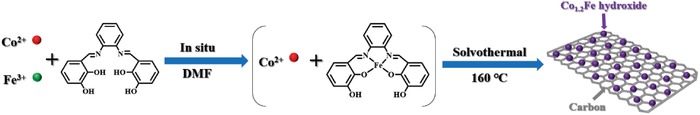
Fabrication process of Co_1.2_Fe/C.

The as‐prepared product was characterized by scanning microscopy (SEM). As shown in Figure S4a,b (Supporting Information), irregular hydroxide nanoparticles were found to embed in amorphous and interconnected carbon matrices. The energy‐dispersive X‐ray spectroscopy (EDX) analysis in Figure S4c (Supporting Information) confirms the coexistence of Co, Fe, C, O, and N elements in the sample. Transmission scanning electron microscopy (TEM) and high‐resolution microscopy (HRTEM) in **Figure** 1a,b provide more details on the uniform dispersion of nanoparticles (marked by blue circles) in carbon matrix with an average size of 4 nm, indicative of a confined growth of hydroxides in this material. Though the boundary between amorphous carbons and CoFe hydroxides could be identified from a zoom‐in image (Figure 1d) of the red rectangular region in Figure 1b, diffraction rings in selected area diffraction (SAED) pattern (Figure 1c) revealed a poor crystalline nature for Fe,Co/C. The poor crystallinity is also evidenced by a featureless XRD pattern in Figure S5 (Supporting Information). The TEM element mapping directly mirrors the homogenous distribution of Co, Fe, C, O, and N in the blend sample (Figure 1e) with an accurate Co/Fe molar ratio of 1.2:1 determined from inductively coupled plasma atomic emission spectroscopy (ICP‐AES) (Table S1, Supporting Information).

**Figure 1:**
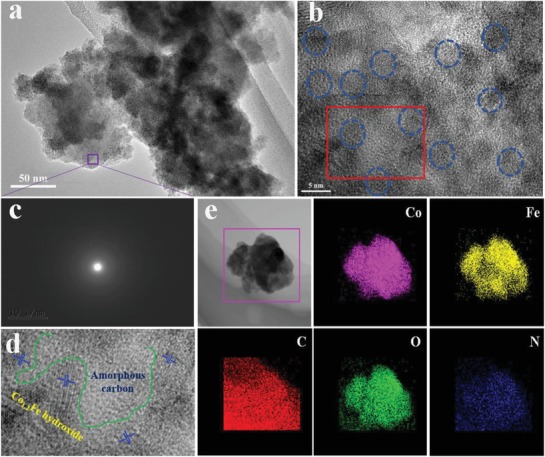
a) TEM and b) HRTEM images of Co_1.2_Fe/C; c) SAED pattern of the marked area in (a); d) The zoom‐in image of the red framed region in (b); e) STEM and the corresponding elemental mapping images of Co_1.2_Fe/C.

X‐ray photoelectron spectroscopy (XPS) was carried out to investigate the composition and chemical oxidation states of the constituent elements in Co_1.2_Fe/C. XPS spectrum in Figure S6a (Supporting Information) shows same element compositions as determined from EDX measurements. The surface molar ratio of Co/Fe in sample was 1.21:1 based on the XPS measurement, which is consistent with the result of ICP analysis. In **Figure** 2a, the XPS spectrum of Fe 2p is featured with two satellite peaks at 718.3 and 733.2 eV, respectively, providing solid evidence to the presence of Fe^3+^.^32^, ^36^, ^37^ The Co 2p spectrum in Figure 2b can be deconvoluted into two spin–orbit peaks at the binding energies of 781.2 (Co 2p_3/2_) and 796.8 eV (Co 2p_1/2_), and the corresponding satellite peaks at 786.1 and 803.1 eV are consistent with the presence of Co^2+^.^37^, ^38^ A prominent peak of 531.3 eV in the O 1s spectrum (Figure 2c) agrees with metal hydroxides as the dominant active species. Peaks at 529.9 and 532.4 eV give clues to the formation of metal–oxygen and carbon–oxygen bonds.^39^ The high‐resolution C 1s peak in Figure 2d can be deconvoluted into four subpeaks arising from the M—C (M = Fe, Co) bonds (283.8 eV), C—C bonds (284.7 eV), C—N/C—O bonds (286 eV), and C=N/C=O bonds, respectively.^32^ The N signal was also verified by the XPS spectrum of N 1s presented in Figure S6b (Supporting Information). Overall, the combined results point to the decomposition of salen‐1 to N‐doped nanocarbons that strongly coupled with CoFe hydroxides.

**Figure 2:**
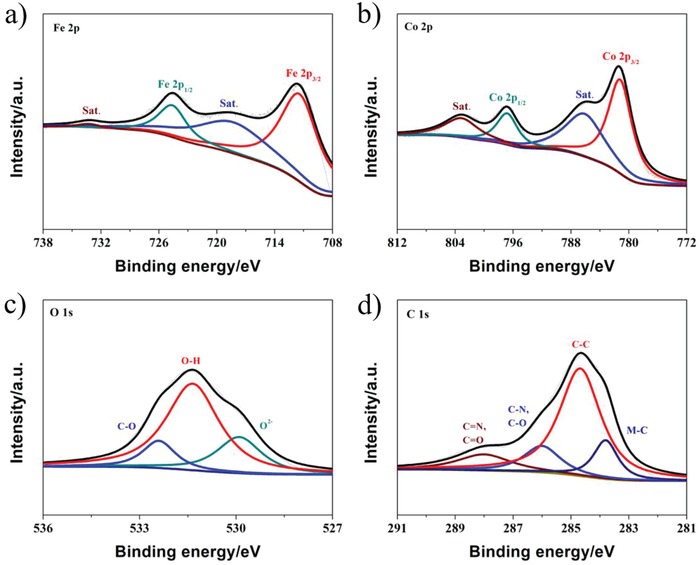
High‐resolution XPS spectra of a) Fe 2p, b) Co 2p, c) C 1s, and d) O 1s for Co_1.2_Fe/C sample.

The fabrication process displayed in Scheme 1 shows the formation of [Fe^III^(salen‐1)]^+^ complex in the stock solution. Evidently, salen‐1 exhibits two intense absorption bands at 285 and 334 nm in DMF due to the π → π* and *n* → π* transition of —C=N— motif,^40^ the red‐shifts occurring for both absorption bands indicate the in situ formation of coordination compound upon the addition of Co^2+^ and Fe^3+^ cations to the above solution (**Figure** 3a). A more solid proof for the molecular identity was based on the electrospray ionization mass spectra (ESI‐MS) analysis of the precursor solution. A signal at *m*/*z* = 475 was found to match the metal–salen species of [Fe^III^(salen‐1)(DMF)]^+^, suggesting preferential coordination of Fe^3+^ with salen‐1 (Figure 3c). However, despite the potential coordination capability of salen‐1 with Co^2+^, there was no evidence for the presence of Co–salen‐1 or FeCo–salen‐1 in DMF. Signals belong to [Co^II^(DMF)_3_]^2+^, [Co^II^(DMF)_4_]^2+^, and [Co^II^(DMF)_2_(NO_3_)]^+^ were identified from MS spectrum, pointing to the solvated Co^2+^ ions (Figure 3c). These findings can also be corroborated by the absorption measurements shown in Figure 3b, where the spectrum of Co^2+^, Fe^3+^, and salen‐1 mixture overlaps with that of Fe–salen‐1 and no characteristic transition band for Co–salen‐1 is shown.

**Figure 3:**
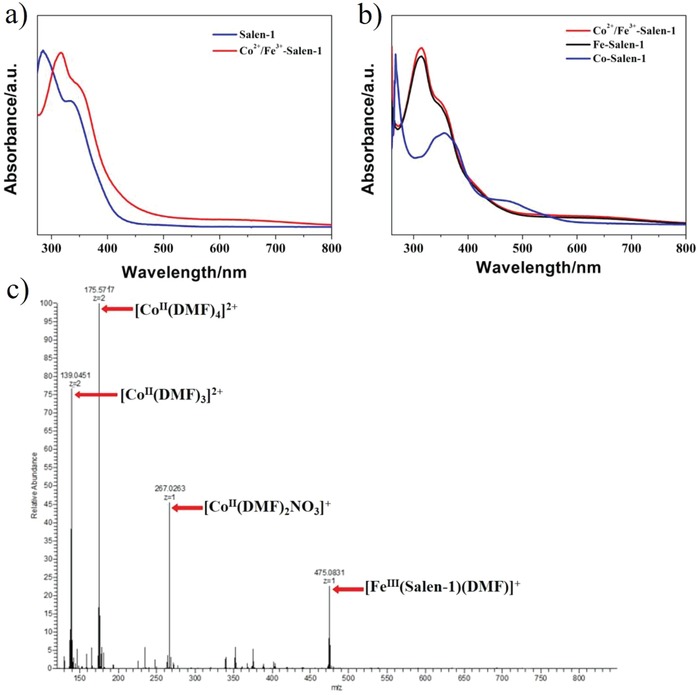
UV–vis absorption spectra of a) Salen‐1 and Co^2+^/Fe^3+^–salen‐1 and b) Co^2+^/ Fe^3+^/salen‐1, Fe–salen‐1 and Co–salen‐1 in DMF; c) ESI‐MS spectrum of the precursor solution in DMF.

Though Fe–salen is the sole molecular precursor in our system, the presence of Co^2+^ is indispensable to the formation of electrocatalyst. Without Co^2+^, no solid product could be produced from the solvothermal reaction. In previous study, cobalt ions have been reported to catalyze the transformation of zeolite imidazole framework‐67 into graphitic‐like carbons.^41^ The co‐existence of Co^2+^ in our system was assumed to play a similar role in facilitating the carbonization of salen ligands.

The electrochemical performance of the in situ synthesized Co_1.2_Fe/C as OER catalyst was assessed in 1 m KOH with a three‐electrode configuration. The working electrode was prepared by drop casting the catalyst onto a rotating disk electrode (RDE) with a mass loading of 0.17 mg cm^−2^, Hg/HgO was employed as the reference electrode and Pt mesh as the counter electrode. To investigate the effect of carbon hybridization, Co_1.2_Fe‐layered double hydroxide (Co_1.2_Fe–LDH) was also prepared by a coprecipitation method (Figure S7, Supporting Information).^42^ **Figure** 4a shows the iR‐corrected linear sweep voltammetry (LSV) curves at a scan rate of 5 mV s^−1^, whereas an overpotential of 260 mV was required by Co_1.2_Fe/C to launch a current density of 10 mA cm^−2^, which is negatively shifted by 50 and 65 mV over that of commercial RuO_2_ and Co_1.2_Fe–LDH. Furthermore, a lower Tafel slope for Co_1.2_Fe/C (45.18 mV dec^−1^) in comparison with Co_1.2_Fe–LDH (90.84 mV dec^−1^) implied rapid reaction kinetics (Figure 4b). The striking difference observed here reveals a notable advantage for integrated carbon in facilitating OER reaction. As one of the most efficient CoFe‐based OER catalysts, the OER activity of Co_1.2_Fe/C is superior to other glassy carbon‐supported CoFe catalysts, such as CoFe_2_O_4_@N–CNFs (*ƞ*_10_ = 349 mV),^26^ Co–Fe–O/rGO (*ƞ*_10_ = 340 mV),^43^ CoFe_2_O_4_/PANI–MWCNTs (*ƞ*_10_ = 310 mV),^44^ H_2_O–plasma exfoliated CoFe LDHs (*ƞ*_10_ = 290 mV),^45^ and Ar–plasma etched CoFe LDHs (*ƞ*_10_ = 266 mV) (Table S2, Supporting Information).^46^

**Figure 4:**
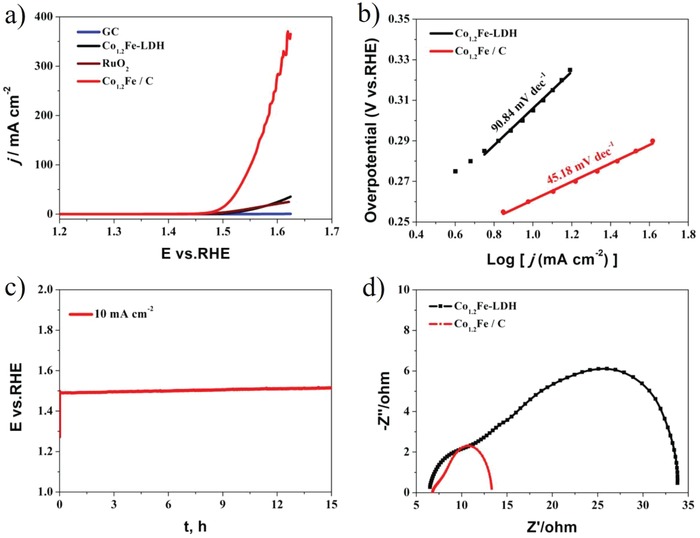
a) LSV curves of glassy carbon (GC), Co_1.2_Fe–LDH, RuO_2,_ and Co_1.2_Fe/C corrected with *i*R compensation in 1 m KOH solution at a scan rate of 5 mV s^−1^; b) Tafel plots of Co_1.2_Fe–LDH and Co_1.2_Fe/C; c) Chronopotentiometric measurement of Co_1.2_Fe/C at 10 mA cm^−2^; d) EIS Nyquist plots of Co_1.2_Fe–LDH and Co_1.2_Fe/C measured under an overpotential of 300 mV.

The durability of Co_1.2_Fe/C at a constant current density of 10 mA cm^−2^ was evaluated by chronopotentiometric measurement. Over a period of 15 h, only slight increase in the potential was observed, demonstrating an outstanding long‐term stability for Co_1.2_Fe/C (Figure 4c). The gaseous products produced by OER reaction were quantitatively determined by gas chromatography with a Faradaic efficiency of 96.5% (Figure S8, Supporting Information).

The OER performance of CoFe/C hybrid was tuned by optimizing the ratios between Co^2+^ and Fe^3+^ cations prior to solvothermal reaction. Although the ratios of Co^2+^:Fe^3+^ were changed, XPS spectra in Figure S9 (Supporting Information) indicate the valence states of +2 for Co and +3 for Fe, and CoFe hydroxides were still the main phase in all resultant Co, Fe/C samples. As demonstrated in Figure S10 (Supporting Information), the highest OER activity was achieved at a Co^2+^/Fe^3+^ ratio of 2:1, consistent with an optimal structure of Co_1.2_Fe/C. The ligand effect on performance was investigated by using a series of salen derivatives (Figure S3, Supporting Information). These ligands were classified into two categories with one category having two phenolic hydroxyl groups (H2salen) and another having four phenolic hydroxyl groups (H4salen). The catalysts derived from H4salen turned out to be more active than those derived from H2salen. On the other hand, replacing the phenylenediamine bridge moiety of salen‐1 with an ethylenediamine bridge, or changing the substitutions on the phenoxyl ring were found to impose negligible effect on the final activities. Although the formation of formal coordination bond between Co^2+^ and —PhO^−^ was not evidently supported, multiple phenolic hydroxyl groups in H4salen are expected to weakly interact with free Co^2+^ ions. These pre‐positioned Co^2+^ might facilitate the subsequent carbonization step.

In order to understand the factors contributing to the high OER activity of metal carbon hybrids, electrochemical impedance spectroscopy (EIS) was carried out for both Co_1.2_Fe/C and Co_1.2_Fe–LDH at an overpotential of 300 mV. As shown in Figure 4d, the first semicircle in the high‐frequency region of Nyquist plot represents the charge transfer resistance from electrolyte to catalyst surface and the second semicircle in low‐frequency region represents the charge transfer resistance inside bulk catalyst.^47^, ^48^ The lower electron transfer resistances for Co_1.2_Fe/C evidenced higher conductivity and faster charge transport of the carbon‐hybridized material.

Besides high electron conductivity, a synergetic effect between CoFe hydroxides and nanocarbons also plays a role in improving catalytic activity. In comparison with Co_1.2_Fe–LDH, the Co 2p_3/2_ XPS peak for Co_1.2_Fe/C is positively shifted from 780.8 to 781.2 eV and the Fe 2p_3/2_ XPS peak for Co_1.2_Fe/C is negatively shifted from 712.1 to 711.6 eV (Figure S11, Supporting Information). The difference in XPS spectra implies partial electron transfer from carbon to metals (Co and Fe) caused by their intimate contact.

Based on the double‐layer capacitance (*C*_dl_), Co_1.2_Fe/C was estimated to have a electrochemical active surface area (ECSA) of 131 cm^−2^ and Co_1.2_Fe–LDH has a ECSA of 214 cm^−2^ (Figure S12, Supporting Information). The ECSA normalized current density (*j*_ECSA_) for Co_1.2_Fe/C is much higher than that for Co_1.2_Fe–LDH (Figure S12d, Supporting Information). Assuming that all Co and Fe ions participate in water oxidation reaction, Co_1.2_Fe/C produced oxygen with a turnover frequency (TOF) of 0.26 s^−1^ at an overpotential of 350 mV. This value exceeds the TOF of Co_1.2_Fe–LDH (0.004 s^−1^) by a factor of 65 at the same overpotential. The above results suggest that the remarkable OER activity for Co_1.2_Fe/C arises from the intrinsic activity of carbon‐supported CoFe nanoparticles rather than the fluctuation on ECSA.

In summary, we have developed a simple, one‐pot method for preparation of electrocatalyst consisting of integrated CoFe hydroxides and nanocarbons from the in situ formed molecular Fe–salen complex and Co^2+^ ion. Due to the catalysis effect of Co^2+^ ion in conversion of salen ligand to amorphous nanocarbons, the hybrid catalyst was fabricated at relatively low temperature, which is a notable advantage over conventional synthetic methods for metal–carbon OER catalysts. The resulting Co,Fe/C showed ligand‐dependent OER activity with the optimized performance obtained by H4salen at a Co/Fe ratio of 2:1. Based on TEM and EIS measurements, the superior OER activity shown here can be dedicated to the following two aspects. First, molecular precursors lead to confined growth of metal hydroxide in carbon matrix, efficiently inhibiting the aggregation of the active sites. Second, the strong coupling between CoFe hydroxides and graphitic carbons improves electron transport in electrode. These results provide a promising molecular approach to rational design of heterogeneous water splitting catalysts under mild conditions.

## Conflict of Interest

The authors declare no conflict of interest.

## Supporting information

Supplementary

## References

[1] M. K. Debe , Nature 2012, 486, 43. 22678278 10.1038/nature11115

[2] D. G. Nocera , Acc. Chem. Res. 2012, 45, 767. 22475039 10.1021/ar2003013

[3] M. Shao , Q. Chang , J. P. Dodelet , R. Chenitz , Chem. Rev. 2016, 116, 3594. 26886420 10.1021/acs.chemrev.5b00462

[4] X. Jia , Y. Zhao , G. Chen , L. Shang , R. Shi , X. Kang , G. I. N. Waterhouse , L. Z. Wu , C. H. Tung , T. Zhang , Adv. Energy Mater. 2016, 6, 1502585.

[5] Y. Zhao , X. Zhang , X. Jia , G. I. N. Waterhouse , R. Shi , X. Zhang , F. Zhan , Y. Tao , L. Z. Wu , C. H. Tung , D. O'Hare , T. Zhang , Adv. Energy Mater. 2018, 8, 1703585.

[6] B. Liu , Y. F. Zhao , H. Q. Peng , Z. Y. Zhang , C. K. Sit , M. F. Yuen , T. R. Zhang , C. S. Lee , W. J. Zhang , Adv. Mater. 2017, 29, 1606521. 10.1002/adma.201606521 28262994

[7] M. G. Walter , E. L. Warren , J. R. Mckone , S. W. Boettcher , Q. X. Mi , E. A. Santori , N. S. Lewis , Chem. Rev. 2010, 110, 6446. 21062097 10.1021/cr1002326

[8] M. W. Kanan , D. G. Nocera , Science 2008, 321, 1072. 18669820 10.1126/science.1162018

[9] Z. Pei , H. Li , Y. Huang , Q. Xue , Y. Huang , M. Zhu , Z. Wang , C. Zhi , Energy Environ. Sci. 2017, 10, 742.

[10] C. C. L. Mccrory , S. Jung , J. C. Peters , T. F. Jaramillo , J. Am. Chem. Soc. 2013, 135, 16977. 24171402 10.1021/ja407115p

[11] Y. Hou , M. Qiu , T. Zhang , J. Ma , S. Liu , X. Zhuang , C. Yuan , X. Feng , Adv. Mater. 2017, 29, 1604480. 10.1002/adma.201604480 27859740

[12] N. Danilovic , R. Subbaraman , K. C. Chang , S. H. Chang , Y. Kang , J. Snyder , A P. Paulikas , D. Strmcnik , Y. T. Kim , D. Myers , V. R. Stamenkovic , N. M. Markovic , Angew. Chem., Int. Ed. 2014, 53, 14016. 10.1002/anie.201406455 25297010

[13] S. Liu , L. Hu , X. Xu , A. A. A. Ghamdi , X. Fang , Small 2015, 11, 4267. 26121217 10.1002/smll.201500315

[14] J. Zhang , Y. Sun , J. Zhu , Z. Gao , S. Li , S. Mu , Y. Huang , Adv. Sci. 2018, 5, 1801375. 10.1002/advs.201801375 PMC6299708 30581716

[15] S. Liu , L. Zheng , P. Yu , S. Han , X. Fang , Adv. Funct. Mater. 2016, 26, 3331.

[16] C. G. Morales‐Guio , L. Liardet , X. Hu , J. Am. Chem. Soc. 2016, 138, 8946. 27344954 10.1021/jacs.6b05196

[17] C. G. Morales‐Guio , M. T. Mayer , A. Yella , S. D. Tilley , M. Grätzel , X. Hu , J. Am. Chem. Soc. 2015, 137, 9927. 26200221 10.1021/jacs.5b05544

[18] J. Wang , W. Cui , Q. Liu , Z. Xing , A. M. Asiri , X. Sun , Adv. Mater. 2016, 28, 215. 26551487 10.1002/adma.201502696

[19] M. Dincǎ , Y. Surendranath , D. G. Nocera , Proc. Natl. Acad. Sci. USA 2010, 107, 10337. 20457931 10.1073/pnas.1001859107 PMC2890790

[20] F. Yu , F. Li , B. Zhang , H. Li , L. Sun , ACS Catal. 2015, 5, 627.

[21] Y. Guo , P. Yuan , J. Zhang , H. Xia , F. Cheng , M. Zhou , J. Li , Y. Qiao , S. Mu , Q. Xu , Adv. Funct. Mater. 2018, 28, 1805641.

[22] B. Yin , X. Cao , A. Pan , Z. Luo , S. Dinesh , J. Lin , Y. Tang , S. Liang , G. Cao , Adv. Sci. 2018, 5, 1800829. 10.1002/advs.201800829 PMC6145217 30250811

[23] S. Han , X. Hu , J. Wang , X. Fang , Y. Zhu , Adv. Energy Mater. 2018, 8, 1800955.

[24] X. Long , J. Li , S. Xiao , K. Yan , Z. Wang , H. Chen , S. Yang , Angew. Chem., Int. Ed. 2014, 53, 7584. 10.1002/anie.201402822 24910179

[25] M. Gong , Y. Li , H. Wang , Y. Liang , J. Z. Wu , J. Zhou , J. Wang , T. Regier , F. Wei , H. Dai , J. Am. Chem. Soc. 2013, 135, 8452. 23701670 10.1021/ja4027715

[26] T. Li , Y. Lv , J. Su , Y. Wang , Q. Yang , Y. Zhang , J. Zhou , L. Xu , D. Sun , Y. Tang , Adv. Sci. 2017, 4, 1700226. 10.1002/advs.201700226 PMC5700636 29201620

[27] B. Xia , Y. Yan , N. Li , H. Wu , X. Lou , X. Wang , Nat. Energy 2016, 1, 15006.

[28] W. Xia , R. Zou , L. An , D. Xia , S. Guo , Energy Environ. Sci. 2015, 8, 568.

[29] T. Y. Ma , S. Dai , M. Jaroniec , S. Z. Qiao , J. Am. Chem. Soc. 2014, 136, 13925. 25216300 10.1021/ja5082553

[30] W. Zhang , X. Jiang , X. Wang , Y. V. Kaneti , Y. Chen , J. Liu , J. S. Jiang , Y. Yamauchi , M. Hu , Angew. Chem., Int. Ed. 2017, 56, 8435. 10.1002/anie.201701252 28382724

[31] J. Wang , H. Zhong , Y. Qin , X. Zhang , Angew. Chem., Int. Ed. 2013, 52, 5248. 10.1002/anie.201301066 23564650

[32] S. Yin , W. Tu , Y. Sheng , Y. Du , M. Kraft , A. Borgna , R. Xu , Adv. Mater. 2017, 29, 1705106.

[33] S. Minakata , T. Ando , M. Nishimula , I. Ryu , M. Komatsu , Angew. Chem., Int. Ed. 1998, 37, 3392. 10.1002/(SICI)1521-3773(19981231)37:24<3392::AID-ANIE3392>3.0.CO;2-G 29711284

[34] E. N. Jacobsen , W. Zhang , M. L. Guler , J. Am. Chem. Soc. 1991, 113, 6703.

[35] L. Yong , J. L. Huang , B. Lian , Y. L. Qian , Chin. J. Chem. 2001, 19, 429.

[36] K. Fan , Y. Ji , H. Zou , J. Zhang , B. Zhu , H. Chen , Q. Daniel , Y. Luo , J. Yu , L. Sun , Angew. Chem., Int. Ed. 2017, 56, 3289. 10.1002/anie.201611863 28194910

[37] X. F. Lu , L. F. Gu , J. W. Wang , J. X. Wu , P. Q. Liao , G. R. Li , Adv. Mater. 2017, 29, 1604437. 10.1002/adma.201604437 27865016

[38] D. Wu , Y. Wei , X. Ren , X. Ji , Y. Liu , X. Guo , Z. Liu , A. M. Asiri , Q. Wei , X. Sun , Adv. Mater. 2018, 30, 1705366. 10.1002/adma.201705366 29333685

[39] M. S. Burke , M. G. Kast , L. Trotochaud , A M. Smith , S. W. Boettcher , J. Am. Chem. Soc. 2015, 137, 3638. 25700234 10.1021/jacs.5b00281

[40] D. Olea‐Román , N. Bélanger‐Desmarais , M. Flores‐Álamo , C. Bazán , F. Thouin , C. Reber , S. E. Castillo‐Blum , Dalton Trans. 2015, 44, 17175. 26374465 10.1039/c5dt02563j

[41] Y. Lin , B. Jia , Y. Fan , K. Zhu , G. Li , C. Y. Su , Adv. Energy Mater. 2017, 7, 1702048.

[42] L. Feng , A. Li , Y. Li , J. Liu , L. Wang , L. Huang , Y. Wang , X. Ge , ChemPlusChem 2017, 82, 483. 10.1002/cplu.201700005 31962033

[43] J. Geng , L. Kuai , E. Kan , Q. Wang , B. Geng , ChemSusChem 2015, 8, 659. 25572639 10.1002/cssc.201403222

[44] Y. Liu , J. Li , F. Li , W. Li , H. Yang , X. Zhang , Y. Liu , J. Ma , J. Mater. Chem. A 2016, 4, 4472.

[45] Y. Wang , Y. Zhang , Z. Liu , C. Xie , S. Feng , D. Liu , M. Shao , S. Wang , Angew. Chem., Int. Ed. 2017, 56, 5867. 10.1002/anie.201701477 28429388

[46] R. Liu , Y. Wang , D. Liu , Y. Zou , S. Wang , Adv. Mater. 2017, 29, 1701546. 10.1002/adma.201701546 28589657

[47] W. Zhang , J. Qi , K. Liu , R. Cao , Adv. Energy Mater. 2016, 6, 1502489.

[48] M. Cao , W. Sheng , Z. Zhuang , Q. Fang , S. Gu , J. Jiang , Y. Yang , J. Am. Chem. Soc. 2014, 136, 7077. 24761994 10.1021/ja502128j

