# Supplementary material for: Iron–Salen Complex and Co2+ Ion‐Derived Cobalt–Iron Hydroxide/Carbon Nanohybrid as an Efficient Oxygen Evolution Electrocatalyst
Source: Adv Sci (Weinh). 2019 Apr 15;6(12):1900117. doi: 10.1002/advs.201900117 (PMC6662268; doi:10.1002/advs.201900117)
Supplement: Supplementary file 1 — Supplementary [file ADVS-6-1900117-s001.pdf]

Copyright WILEY-VCH Verlag GmbH & Co. KGaA, 69469 Weinheim, Germany, 2019.

ADVANCED SCIENCE
Open Access

# Supporting Information

for *Adv. Sci.,* DOI: 10.1002/advs.201900117

# Iron–Salen Complex and $Co^{2+}$ Ion-Derived Cobalt–Iron Hydroxide/Carbon Nanohybrid as an Efficient Oxygen Evolution Electrocatalyst

*Jian Du, Guoquan Liu, Fei Li,* Yong Zhu, and Licheng Sun*

Supporting Information

# Iron-salen complex and $Co^{2+}$ ion derived cobalt-iron hydroxide/carbon nanohybrid as an efficient oxygen evolution electrocatalyst

*Jian Du+, Guoquan Liu+, Fei Li,* Yong Zhu and Licheng Sun*

**Materials**

All chemicals and solvents were commercially available and used without further purification. Cobaltous nitrate hexahydrate ($Co(NO_3)_2{\cdot}6H_2O$, 99.99%), Iron nitrate nonahydrate ($Fe(NO_3)_3{\cdot}9H_2O$, 99.99%), o-phenylenediamine ($C_6H_8N_2$, 99.5%), Ethylenediamine($C_2H_8N_2$, 99.5%), 2-hydroxybenzaldehyde ($C_7H_6O_2$, 99%), 2,3-dihydroxybenzaldehyde ($C_7H_6O_3$, 97%), 3-methoxy-2-hydroxybenzaldehyde ($C_8H_8O_3$, 99%), 3-nitro-2-hydroxybenzaldehyde ($C_7H_5NO_4$, 98%), Sodium carbonate anhydrous ($Na_2CO_3$, 99.999%) and potassium hydroxide (KOH, 95%) were purchased from Aladdin chemical company. Sodium hydroxide (NaOH, 96%) were purchased from Alfa Aesar. Nafion solution (5wt%) was obtained from Sigma-Aldrich. Methanol ($CH_3OH$), Ethanol ($C_2H_5OH$) and N, N-dimethylformamide (DMF) were analytical pure and purchased from commercial supplier.

**Synthesis of N, N'-bis-(2,3-dihydroxybenzylididene)-o-phenylenediamine (Salen-1)**

The salen-1 ligand was synthesized according to a reported procedure with minor modification.[1] Generally, methanol solution (50 ml) containing o-phenylenediamine (1 mmol) and 2,3-dihydroxybenzaldehyde (2 mmol) was refluxed and heated at 70 ℃ for 2h. After evaporating part of the solvent, the crystallization was induced by the addition of a small amount of deionized water. Afterward, the crude product was separated from the mixture by filtration and washed with $CH_3OH$ several times to get rid of the impurities. The resultant salen complex was finally obtained after vacuum drying, and the corresponding EIS-MS and $^1H$ NMR spectra were presented in Figure S1. EIS-MS ($CH_2Cl_2$, m/z): 347 [M-H]; $^1H$

NMR (500 MHz, $CD_3OD$): δ 12.88 (s, 2H), 9.23 (s, 2H), 8.88 (s, 2H), 7.42 (m, 4H), 7.12 (d, 2H), 6.94 (d, 2H), 6.79 (t, 2H).

**Synthesis of other salen ligands (Salen-2 to 5)**

These salen ligands were synthesized as described above except the difference in the feed material, and the corresponding EIS-MS spectra were presented in Figure S2.

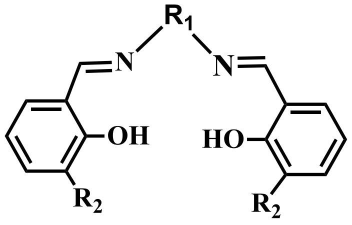


| | | | |
|---|---|---|---|
| $R_1$: Ethylenediamine | $R_2$:-OH | M = 300 | **salen-2** |
| $R_1$: o-phenylenediamine | $R_2$: $-NO_2$ | M = 406 | **salen-3** |
| $R_1$: o-phenylenediamine | $R_2$: -H | M = 316 | **salen-4** |
| $R_1$: o-phenylenediamine | $R_2$: $-OCH_3$ | M = 376 | **salen-5** |

**Preparation of $Co_{1.2}Fe$ /C and $Co_{1.2}Fe$ LDH electrocatalysts**

$Co_{1.2}Fe$ /C hybrid was synthesized by the method of solvothermal carbonization. Briefly, 87 mg salen-1 complex was dissolved in 10 ml DMF to form solution A. 145.5 mg $Co(NO_3)_2{\cdot}6H_2O$ and 101 mg $Fe(NO_3)_3{\cdot}9H_2O$ were dissolved in 10 ml DMF to form solution B. The molar ratio of salen/$Co^{2+}$/$Fe^{3+}$ was 1:2:1. Solution B was then poured into solution A with the color changing from red to deep green. The resulting solution was transferred into a 50 ml of stainless-steel Taflon-lined autoclave and heated at 160 °C for 12 h in an electrical oven. A solid product was obtained by filtration and washed with ethanol for several times. The $Co_{1.2}Fe$/C sample was obtained after the solid powder dried at 60 °C for several hours. Other samples with different $Co^{2+}$/$Fe^{3+}$ molar ratios were also prepared by the same method.

The other CoFe/C sample were fabricated by hydrothermal carbonization of salen 2-5 as described above, which denoted as Salen-2, Salen -3, Salen -4 and Salen -5, respectively. Their OER activity were determined by LSV curves showed in Figure S3, indicating that the salen ligands with four phenolic hydroxyl groups were more promising as the precursor for preparing metal-carbon hybrid electrocatalysts towards water oxidation reaction.

According to a previously reported literature, [2] $Co_{1.2}Fe$ layer double hydroxides were synthesized by a co-precipitation reaction between a metal salt aqueous solution and a $Na_2CO_3$ and NaOH mixed solution.

**Material characterization**

$^1$H NMR spectra was collected by Bruker AVANCE III 500 instrument. Electrospray ionization mass spectra (EIS-MS) was obtained by Q-Tof Micromass spectrometer. UV-vis spectra were collected on an Agilent 8453 diode array spectrophotometer. The morphologies of the prepared samples were observed by Nova NanoSEM 450 equipment. The microstructure and lattice fringe were characterized by TEM (FEI TF30). X-ray diffraction (XRD) patterns were acquired on a D/max-2400 diffractometer (Japan Rigaku Rotaflex) with Cu Kα radiation (λ = 154.1 Å) to determine the crystal structures. The chemical composition and element states of the samples were confirmed by X-ray photoelectron spectroscopy (XPS) (Thermo Scientific ESCALAB250). The binding energy (BE) was calibrated with respect to the C 1s peak at 284.6 eV. Raman spectroscopy were collected on a DXR Smart Raman using $Ar^+$ laser excitation. The quantitative analysis of metal elements in the samples were conducted using Inductively Coupled Plasma-Atomic Emission (ICP-AES) spectrometer (Optima 2000DV, America PerkinElmer Corp.).

**Electrode preparation**

The catalyst ink was prepared by dispersing 5 mg of the catalyst into 1ml of ethanol/water (450/500 μl) mixed solution containing 50 μl 5% Nafion, the mixture was then ultrasonicated for 30 min to become homogeneous. The rotating disk electrode (RDE) made of glassy carbon (5 mm diameter, 0.196 $cm^2$) was polished using alumina powder on felt polishing pads prior to use. Subsequently, 12 μl of the ink was dropped on the surface of the glassy carbon with an overall catalyst loading of ~ 0.174 mg $cm^{-2}$. Finally, the resulting catalyst film was dried at 60 ℃ for electrochemical measurements.

**Electrochemical measurements**

Electrochemical measurements were carried out in a three-electrode system on CHI 660E Electrochemical Analyzer (Shanghai Chenhua Instrument Co., LTD) at room temperature in 1 M KOH aqueous solution. The RDE decorated with catalyst film was employed as working electrode, Pt mesh and HgO/Hg electrode were used as counter and reference electrodes, respectively. All potentials reported here were converted to the reversible hydrogen electrode (RHE) scale with $E_{RHE} = E_{HgO/Hg} + 0.0592$ pH + 0.12 V.

Before electrochemical measurements, the working electrode was activated by a chronoamperometry scan until a stable I-t curve obtianed. Liner sweep voltammetry (LSV) curves corrected with iR-compensation were tested at a scan rate of 5 mV $s^{-1}$. Tafel slopes were determined by cyclic voltammetry with a scan rate of 1 mV $s^{-1}$ to reduce the capacitance and were calculated by plotting overpotential against Log (current density). Chronopotentiometry curve was obtained at the applied current density of 10 mA $cm^{-2}$. The electrochemical impedance spectroscopy (EIS) was performed at an overpotential of 300 mV at the amplitude of the sinusoidal voltage of 5 mV over a frequency range from 0.1 Hz to $10^5$ Hz. The double-layer capacitance ($C_{dl}$) measured by cyclic voltammetry at different scan rates (10 to 60 mV $s^{-1}$ with an interval of 10 mV $s^{-1}$) in a voltage range of 1.125 ~1.225 V (non-faradaic region) was used to estimate the electrochemical active surface area (ECSA). By

plotting the capacitive current density ($j_{anodic}$ -$j_{cathodic}$) against the scan rate, the value of $C_{dl}$ can be determined as half of the slope.

The TOF values of catalysts deposited on glassy carbon were calculated by assuming that every metal atom is involved in the catalysis:

$$\text{TOF} = \frac{j \cdot A}{4 \cdot F \cdot n}$$

Where j is the current density obtained at overpotential of 350 mV, A is the surface area of the glassy carbon (0.196 $cm^{-2}$), F is the Faraday constant (96485 C $mol^{-1}$) and n is the moles of the metal atom loaded on the electrode.

To determine the faradic efficiency, a home-made single compartment gas-tight cell was sealed and purged with Ar for 1h. The electrolysis was performed under a constant current of 1 mA for 2h, and the actual amount of $O_2$ in the headspace was quantified by gas chromatography every 20 min. The Faradic efficiency was calculated from the total amount of charge passed through the cell (Q) and the total amount of oxygen produced ($nO_2$) according to the equation, Faradaic efficiency = 4F × $nO_2$/Q, where F is the Faraday constant (96485 C $mol^{-1}$).

Reference

[1] F. Mohanders, M. S. Niasari, *New J. Chem*, **2014**, *38*, 4501.

[2] L. Feng, A. Li, Y. Li, J. Liu, L. Wang, L. Huang, Y. Wang, X. Ge, *ChemPlusChem* **2017**, *82*, 483.

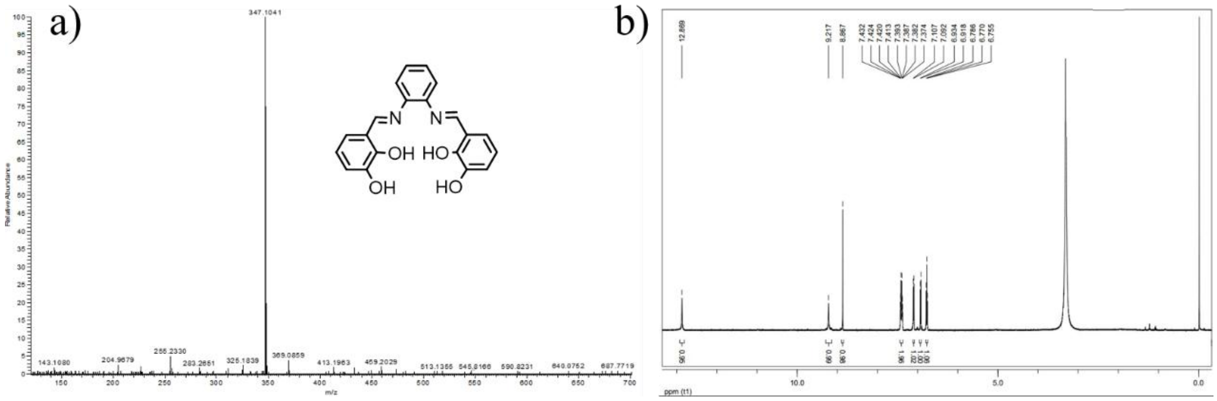


**Figure S1.** (a) EIS-MS spectrum of Salen-1 in $CH_2Cl_2$; (b) $^1H$ NMR spectrum of Salen-1 in $CD_3OD$.

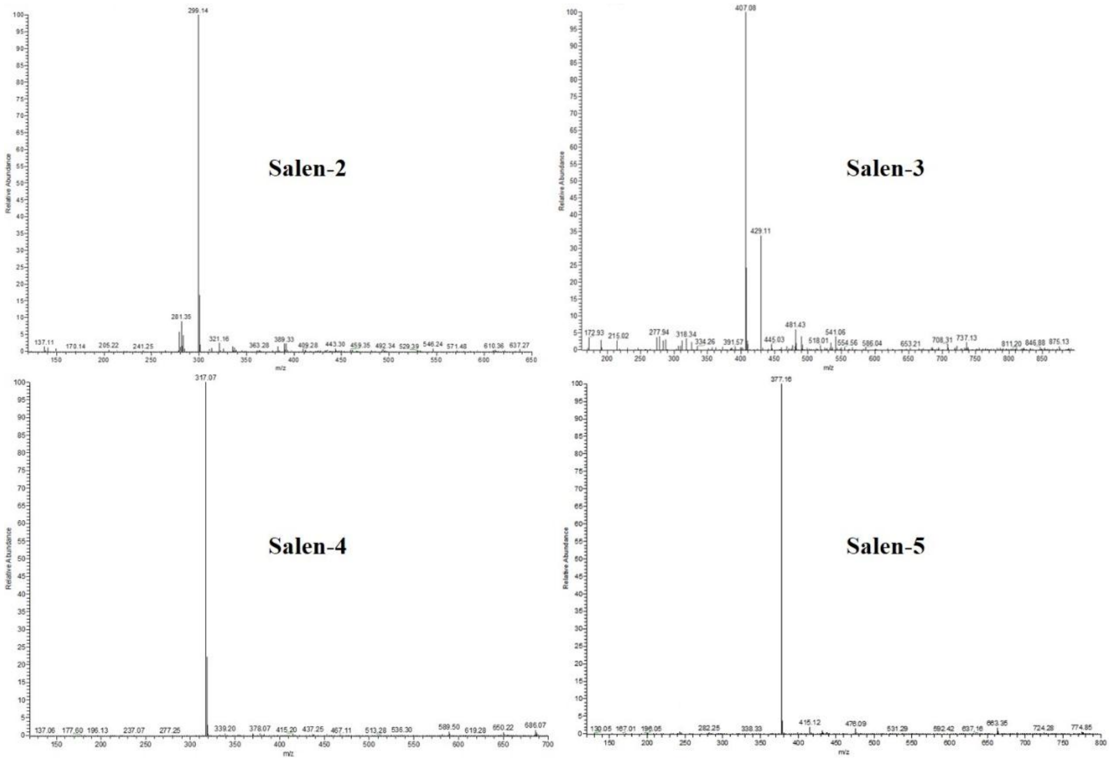


**Figure S2.** (a-e) EIS-MS spectra of salen ligands in $CH_2Cl_2$.

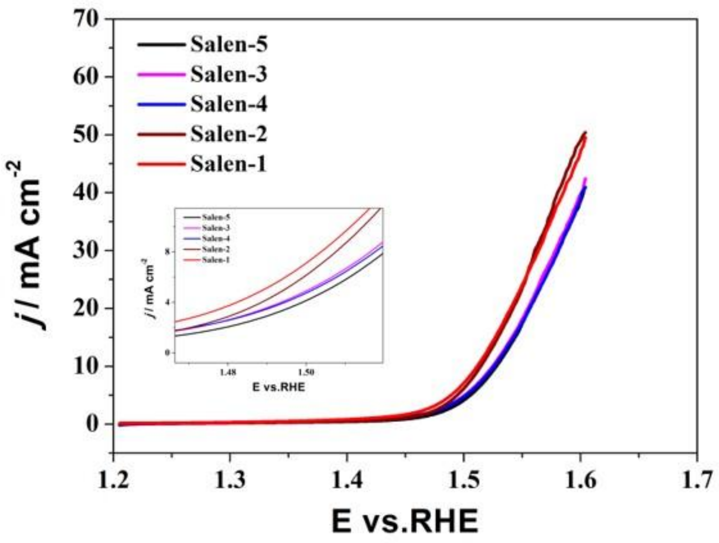


**Figure S3.** LSV curves of Salen derived Fe, Co/C samples loaded on GC (diameter: 3 mm) in 1 M KOH without iR compensation.

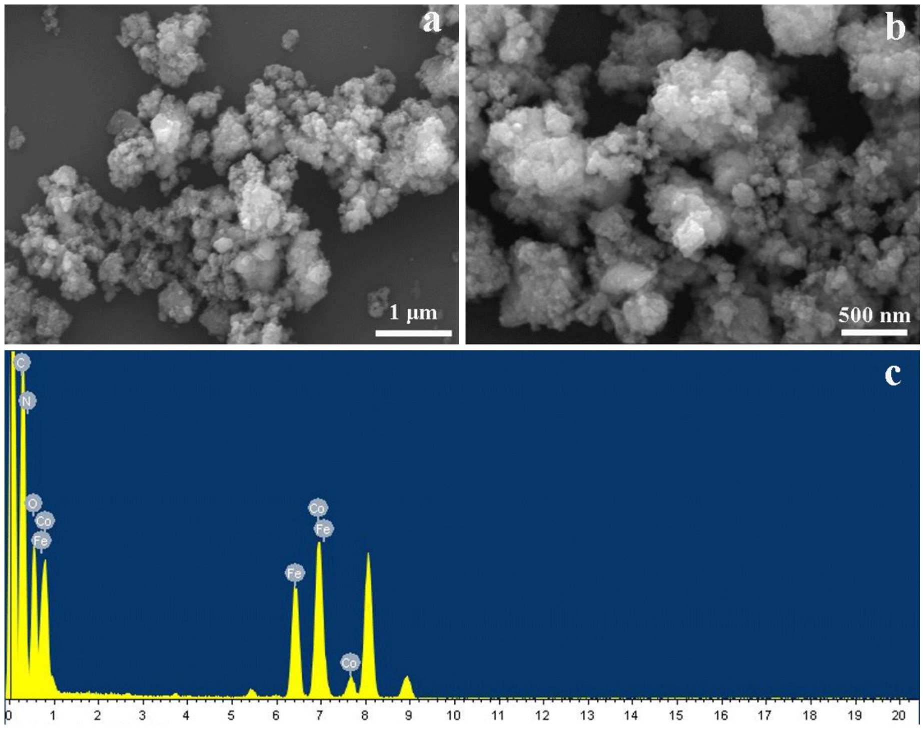


**Figure S4.** (a-b) SEM images and (c) EDX analysis of $Co_{1.2}Fe/C$.

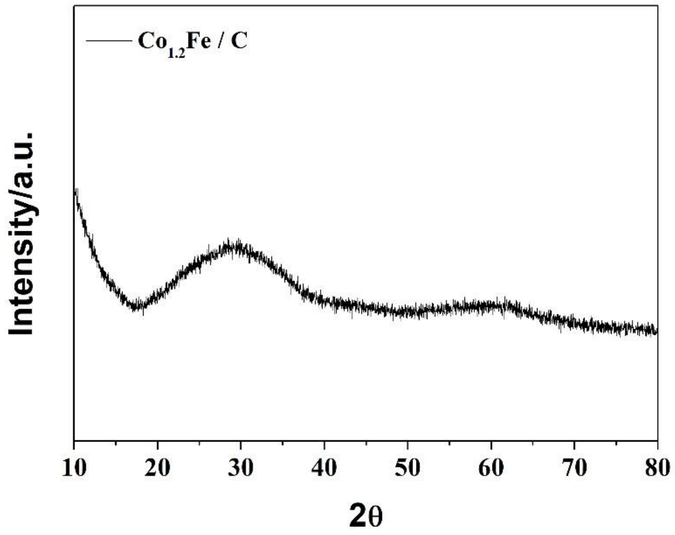


**Figure S5.** XRD pattern of $Co_{1.2}Fe/C$.

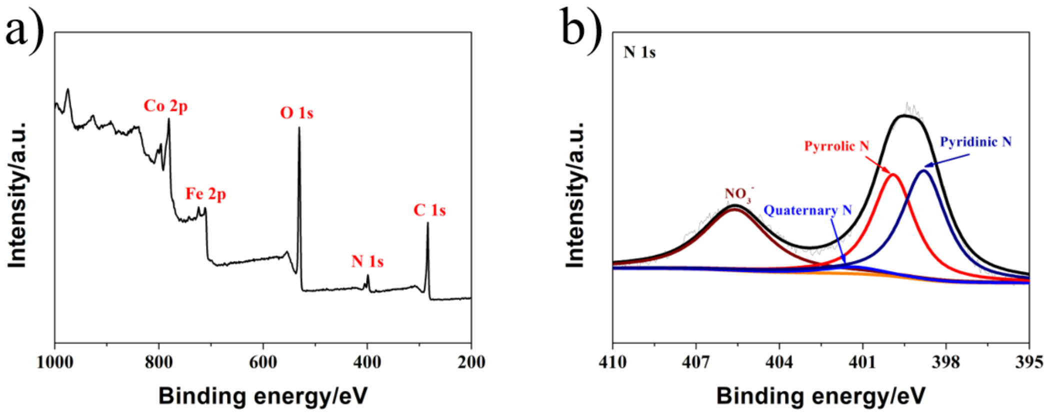


**Figure S6.** (a) XPS survey spectrum of $Co_{1.2}Fe/C$ and (b) high-resolution XPS spectrum of N 1s.

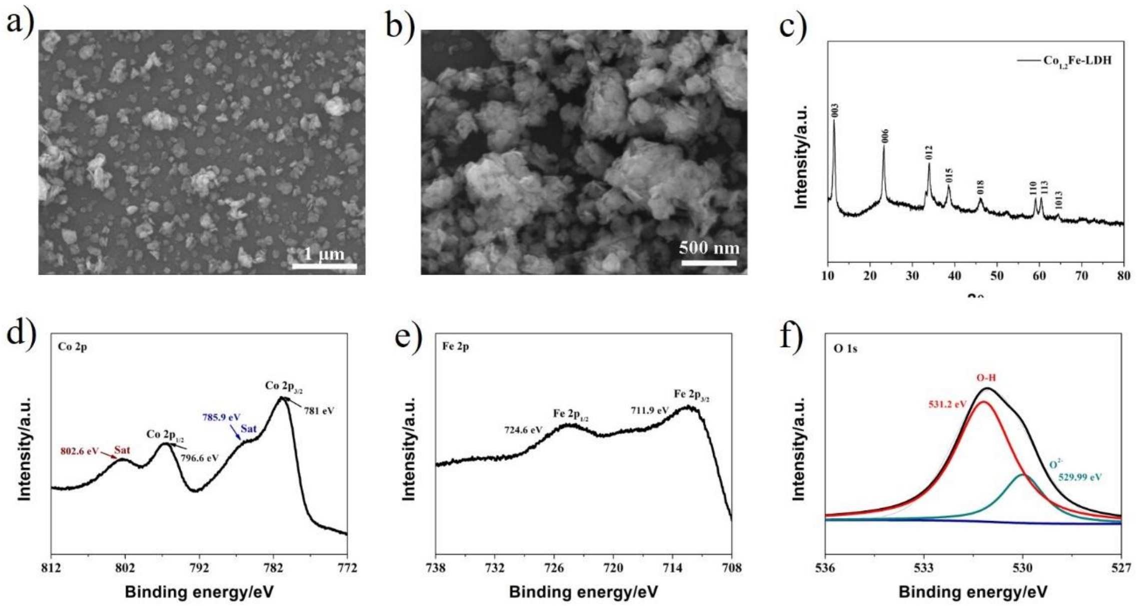


**Figure S7.** (a-b) SEM images and (c) XRD pattern of $Co_{1.2}Fe$-LDH, the observed diffraction peaks located at 11.48º, 23.26º, 34.04º, 38.52º, 46.08º, 59.08º, 60.44º and 64.42º are indexed to the (003), (006), (012), (015), (018), (110), (113) and (1013) planes of CoFe-LDH (JCPDF NO. 50-0235); high-resolution XPS spectra of (d) Co 2p, (e) Fe 2p and (f) O1s.

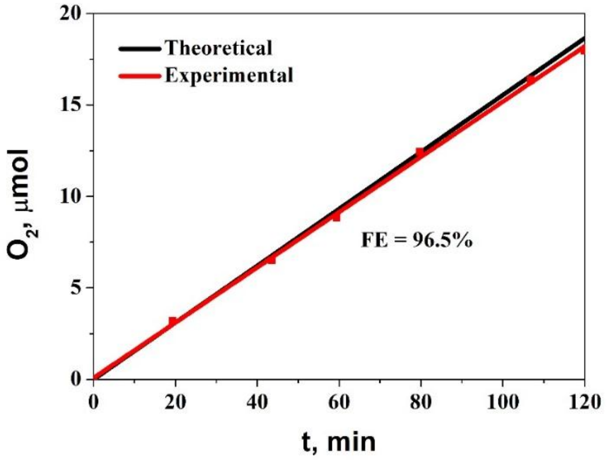


**Figure S8.** Determination of Faradaic efficiency. The experimental and theoretical $O_2$ evolution amount were obtained by electrolysis of $Co_{1.2}Fe/C$ at the constant oxidative current of 1 mA for 2h. The Faradaic efficiency was calculated to be 96.5%.

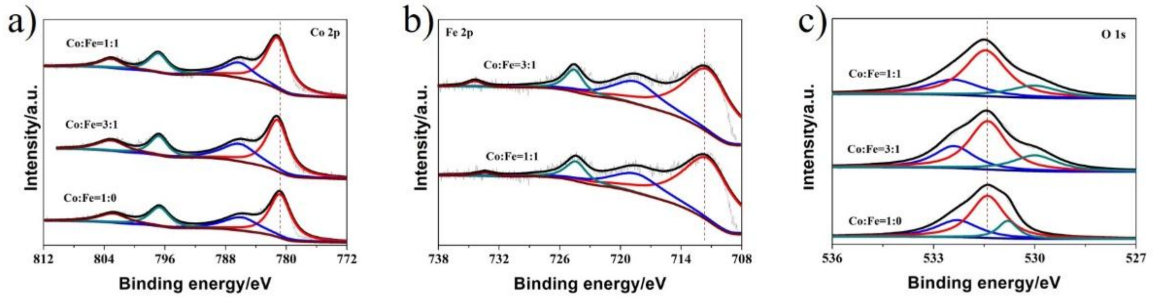


**Figure S9**. High resolution XPS spectra of (a) Co 2p, (b) Fe 2p and (c) O 1s for Co, Fe/C samples with different ratios of Co/Fe (1:0, 1:1 and 3:1). According to the Co 2p and Fe 2p spectra, the valence states were +2 for Co and +3 for Fe for all resultant samples. In addition, the prominent O 1s signals at 531.5 eV manifest that hydroxides were the main species in all three Co, Fe/C samples.

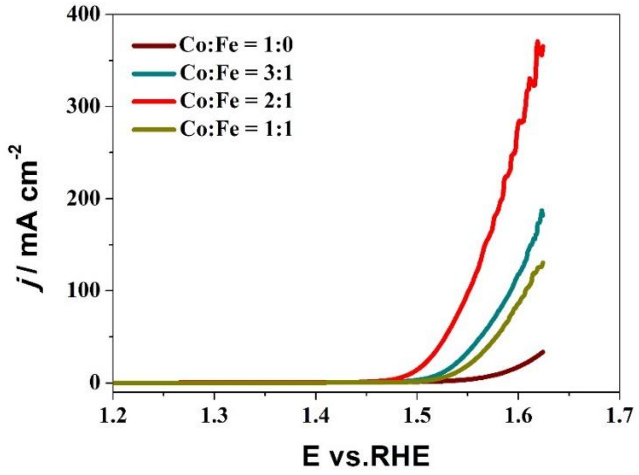


**Figure S10.** LSV curves of Salen derived Fe, Co /C samples with different ratios of $Co^{2+}/Fe^{3+}$.

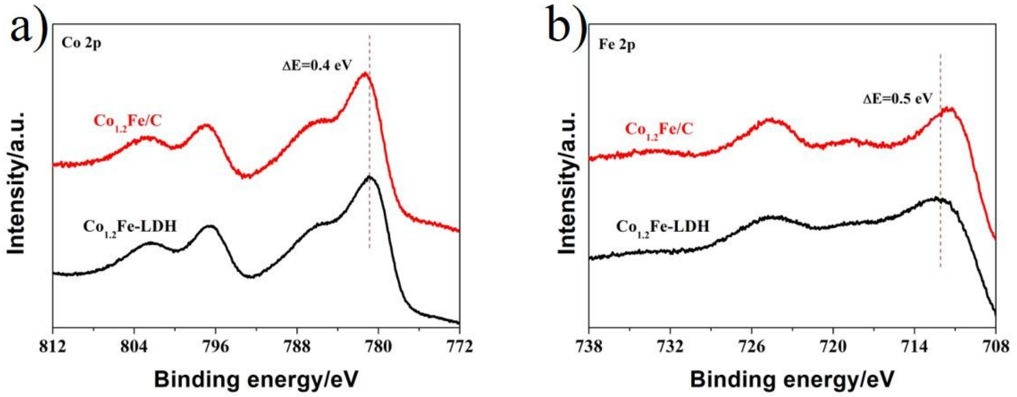

**Figure S11**. (a) Co 2p and (b) Fe 2p XPS spectra of $Co_{1.2}Fe$-LDH and $Co_{1.2}Fe/C$.

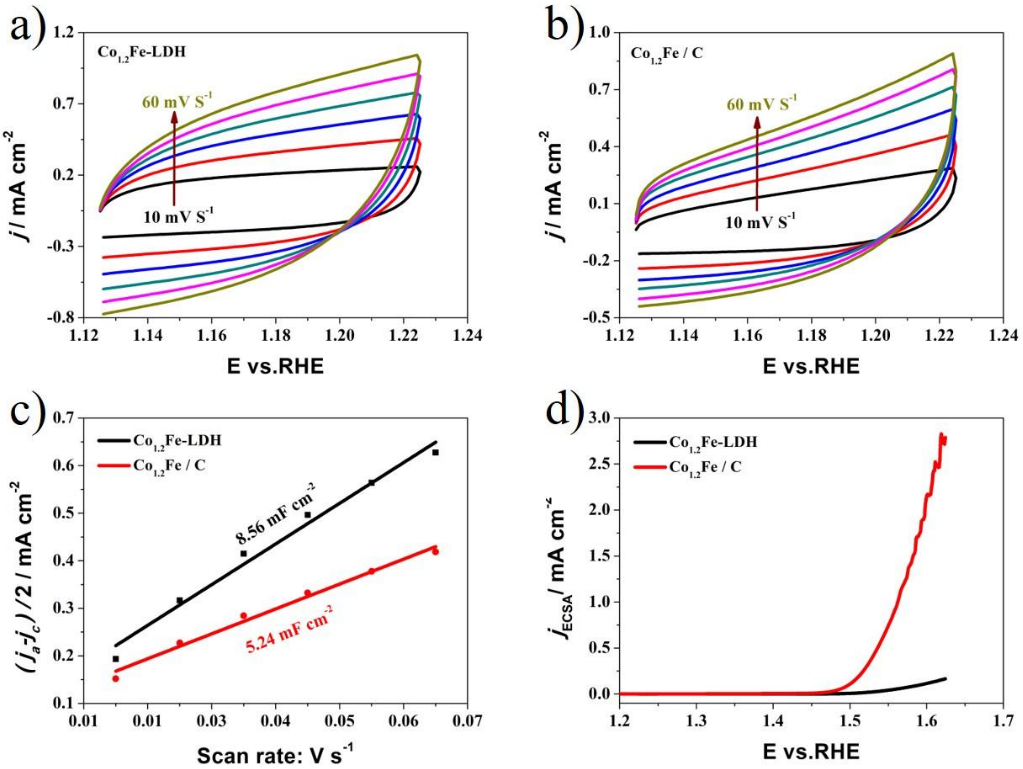


**Figure S12.** CV curves of (a) $Co_{1.2}Fe$-LDH and (b) $Co_{1.2}Fe/C$ at various scan rates (10 to 60 mV $s^{-1}$ with an interval of 10 mV $s^{-1}$) within the potential range of 1.13~1.23 V. (c) Charging current density difference at 1.18 V plotted against scan rate for $Co_{1.2}Fe$-LDH and $Co_{1.2}Fe/C$. (d) LSV curves of $Co_{1.2}Fe$-LDH and $Co_{1.2}Fe/C$ normalized by ECSA.

**Table S1**. The molar ratio of Co/Fe in bulk composition.

| | Mass concentration (mg $L^{-1}$) | Molar concentration (μmol) | Molar ratio |
|---|---|---|---|
| Co | 21.02 | 0.356 | 1.2:1 |
| Fe | 16.83 | 0.3 | |

**Table S2.** OER performance of $Co_{1.2}Fe/C$ and the recently reported CoFe-based electrocatalysts.

| Electrocatalyst | Electrolyte | $\eta_{(10\ mA\ cm-2)}$ / mV | Tafel slope: mV/dec | References |
|---|---|---|---|---|
| **$Co_{1.2}Fe/C$** | **1M KOH** | **260** | **45.18** | **This work** |

| | | | | |
|---|---|---|---|---|
| CoFe LDH-Ar | 1M KOH | 266 | 37.85 | Angew. Chem. Int. Ed. 2017, 56, 5867 |
| $H_2O$-Plasma Exfoliated CoFe LDHs | 1M KOH | 290 | 36 | Adv. Mater. 2017, 29, 1701546 |
| CoFe LDH | 1M KOH | 300 | 40 | ACS Appl. Mater. Interface 2016, 8, 34474 |
| $CoFe_2O_4$/PANI-MWCNTs | 1M KOH | 310 | 30.69 | J. Mater. Chem. A 2016, 4, 4472 |
| Fe-CoOOH/G | 1M KOH | 330 | 37 | Adv. Energy Mater 2017, 1602148 |
| CoFeOx | 1M KOH | 330 | NA | J. Am. Chem. Soc. 2015, 137, 9927 |
| Co-Fe-O/rGO | 1M KOH | 340 | 31 | ChemSusChem. 2015, 8, 659 |
| $CoFe_2O_4$@N-CNFs | 1M KOH | 349 | 75 | Adv. Sci. 2017, 4, 1700226 |
